# An Implanted Closed-loop Chip System for Heart Rate Control: System Design and Effects in Conscious Rats^☆^

**DOI:** 10.1016/S1674-8301(10)60018-8

**Published:** 2010-03

**Authors:** Yuxuan Zhou, Yuan Yuan, Juan Gao, Ling Yang, Feng Zhang, Guoqing Zhu, Xingya Gao

**Affiliations:** a Department of Biomedical Engineering, Nanjing Medical University, Nanjing, 210029, China; b Department of Physiology, Nanjing Medical University, Nanjing, 210029, China

**Keywords:** closed-loop regulation, implanted chip system, set point, heart rate, vagus nerve stimulation, Remote-controlled animal

## Abstract

**Objective:**

To evaluate the efficiency of an implanted chip system for the control of heart rate (HR).

**Methods:**

The HR was recorded in six conscious Sprague-Dawley (SD) rats. An implanted chip system was designed to regulate the HR by stimulating the right cervical vagus nerve according to the feedback of real time HR. Each rat was subjected to 30-min regulation and 30-min recovery. The change of HR during the regulation period was compared with the control. The ECG was recorded during the experiment for 24 h.

**Results:**

The ECG signals were successfully recorded during the experiment. The HR was significantly decreased during the period of regulation compared with control (-79.3 ±34.5, *P* < 0.01, *n* = 6) and then recovered to normal after regulation.

**Conclusion:**

The described implanted chip system can regulate the HR to a designated set point.

## INTRODUCTION

Medication is most commonly used to treat such cardiovascular conditions as hypertension, heart failure, and arrhythmias. However, these drugs have many side effects. For example, the conventional medication for hypertension may lead to orthostatic hypotension, arrhythmias and water and electrolyte disorders [1]. Using nitrates to treat heart failure may result in headache, hypotension and dizziness [2]. Resistance may develop to many of these drugs. Furthermore, almost all of them need to be taken for life and this is hard for most patients to accept. A non-pharmacotherapy method to solve these problems is highly desirable.

Implantable devices provide a feasible alternative in such cases [3]–[6]. Instead of taking medicine every day, the patients may prefer receiving an implanted device to relieve the symptoms for a relatively long time. Actually the developments in therapeutic and diagnostic devices have increasingly improved the patients' quality of life. The rapid development of microelectronics technology improves the possibility of the clinical application of implantable devices. The implantable device has become a research focus in the biomedical field, and both medical researchers and physicians are realizing that such devices may be a new therapy for some disorders that are resistant to drug therapy.

Tachycardia is a common clinical arrhythmia, and vagus nerve stimulation (VNS) may be a potential way to cure it. VNS has been proposed as a possible treatment in several conditions, e.g. myocardial infarction (MI) [7], atrial fibrillation (AF) [8] and angina pectoris [9]. It has also proved to be an effective way to influence heart rate in both animals and humans [10]–[11]. An implanted vagus nerve stimulator could work in synergy with β-blockers and ACE inhibitors, by enhancing their life-saving action [12]. However, the human body is a complex closed-loop regulation system. In patients with dysfunctional control mechanisms, the general open-loop device is not safe or effective when interacting with the circulatory system [4],[5],[13]. Therefore, an Implanted Closed-loop Chip System was developed to control the circulatory function and cure rapid arrhythmias. In our past studies the implanted chip system has been successful in controlling blood pressure in both SD rats and spontaneously hypertensive rats (SHR) [4],[5],[14]. In order to regulate the HR smoothly and safely, a similar implantable closed-loop chip system was designed. The chip system function included collecting heart rate signals, performing data analysis, providing vagus nerve stimulation and maintaining external wireless communication. The purpose of the present study was to employ the chip in vivo, and to determine its efficiency in regulating heart rate in normal conscious rats.

## MATERIALS AND METHODS

### System Description

**Fig. 1:**
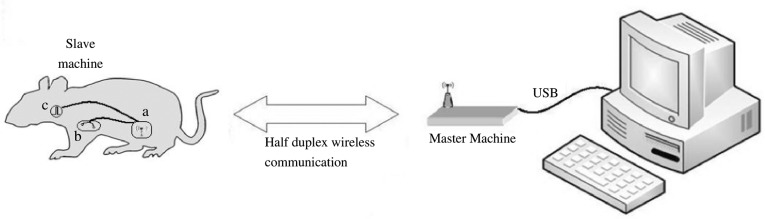
System diagram. a:Recording ECG, HR; b:ECG electrodes; c.stimulation.

The whole system consisted of the slave machine (ECG sensing electrodes, stimulation electrodes, amplifiers, a processor, a wireless transceiver and a silver-zinc battery), the master machine (processor, a wireless-transceiver and a USB bridge circuit) and PC-based user software (for displaying ECG signals and controlling the slave machine). The slave machine detected the ECG signals from the two precordial electrodes. The signals were amplified with a custom amplifier and fed to an on-chip 10-bit A/D convertor sampling at 500 Hz. The digital ECG data were sent to the master machine by the Nordic wireless transceiver NRF24L01. On the master side, another wireless transceiver collected the signals and sent back commands by way of half duplex communication. A MSP430 processor was used for buffering the data, formatting the chip, and communicating with a PC by a USB bridge circuit (***Fig. 1***). The slave machine was implanted subcutaneously in the abdomen. The stimulation electrodes were placed around the right cervical vagus nerve, and the precordial recording electrodes were located subcutaneously. The ECG was detected and analyzed by the chip. Depending on the instantaneous ECG R-R intervals, the system generated corresponding frequency electric pulses to stimulate the vagus nerve. At the same time through wireless transmission, host computer software received and displayed the real-time ECG waveforms and cardiac cycle. In addition, 24-h data was also stored in a flash memory of the chip.

#### The Slave Machine

##### The Processor

A minitype processor was used as the MPU of the slave machine, which allowed the chip to be small enough to be implanted in a rat. The processor had two timers: Timer A and a Watchdog Timer. Timer A was assigned to control the frequency and stimulation pulse width, while the Watchdog Timer contributed to the sampling rate of the A/D convertor for ECG signal collecting[15],[16]. The real time results of the A/D convertor were sent wirelessly through the Nordic NRF24L01 to the master machine and the average cardiac cycles were saved to the flash memory at the same time. Thus the intact heart rate changing trend could be conserved during the experiment to avoid some accidental wireless communication corruptions. Almost 30 h of data were stored in the flash memory of the slave machine and could be collected afterwards through a user command by the master machine.

##### The Amplifier

**Fig. 2:**
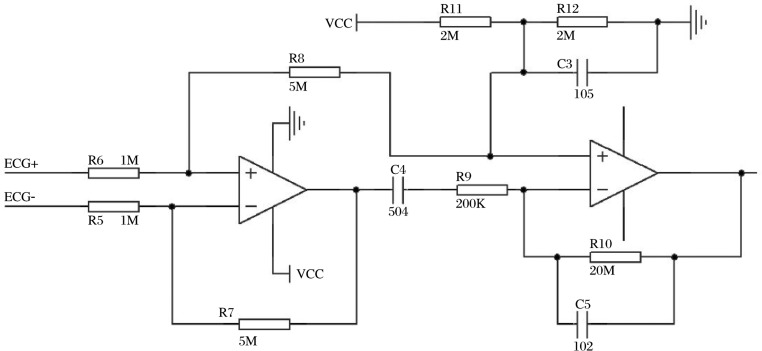
ECG amplifier schematics

In order to achieve a minimum component count and smaller size, we used one rail-to-rail I/O dual Operational Amplifier (OPA2369, Texas Instruments, USA) to build a differential amplifier (***Fig. 2***). The output voltage of the circuit could be calculated by 
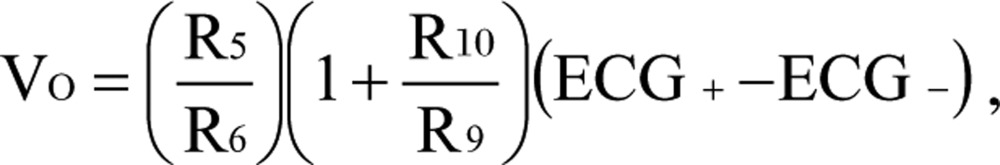
 so the gain here was 54 dB. A voltage division circuit composed of R_11_, R_12_ and C_3_ was designed to raise the baseline of the amplifier output in order to fit the on-chip A/D convertor. The pass-band of the circuit was 1.6 Hz-40 Hz.

##### R-R Interval Recognition

As a closed-loop system, stimulation could be dynamically adjusted according to the real-time heart rate (HR). Continuously acquired ECG data were used to recognize the R-R intervals. The absolute value of 9-point difference data was compared with the threshold. When the result was positive, an R wave was recognized. Still while an R wave was recognized, a refractory timer was set. Thus, during the refractory period no other difference data would be recognized as an R wave until the timer ended in order to avoid repeated error recognition of the same R wave. The refractory timer could be set according to the different experimental animals. Obviously the real-time HR could be calculated by the measurement of the cardiac cycle.

##### Stimulation Method

**Fig. 3:**
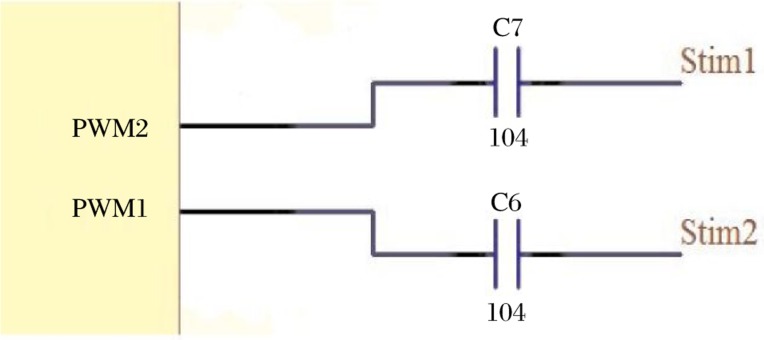
PWM stimulation schematics.

**Fig. 4:**
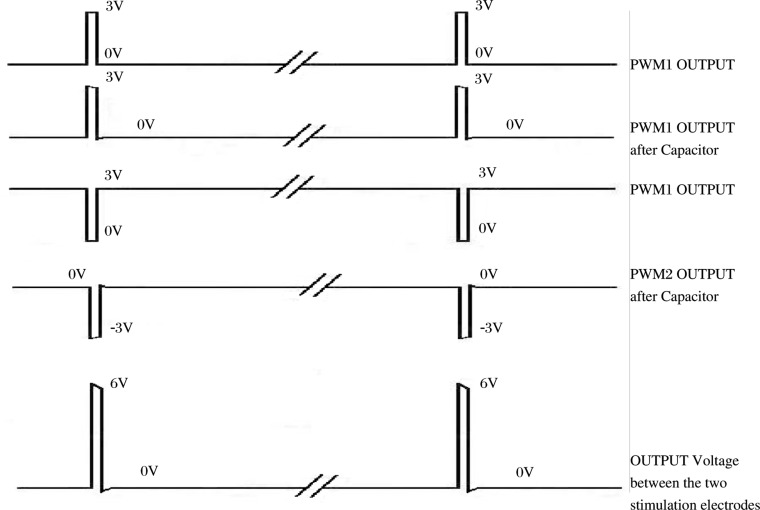
The pattern of stimulus changing.

The stimulation part of the system was controlled by the PWM generator of the chip. As we intended to produce a 6V stimulation with a 3V power supply, the circuit was designed as follows. A differential voltage of 6V was achieved due to the 2 PWM outputs (***Fig. 3***). As illustrated in ***Fig. 4***, when a pulse occurred one of the PWM pulses was +3V and the other one was -3V (after the capacitor), hence the differential voltage between the two stimulation electrodes reached 6V. The two capacitors ensured the voltage became zero after the pulse so that there was no any direct current between the two electrodes, avoiding tissue electrolysis.

**Fig. 5:**
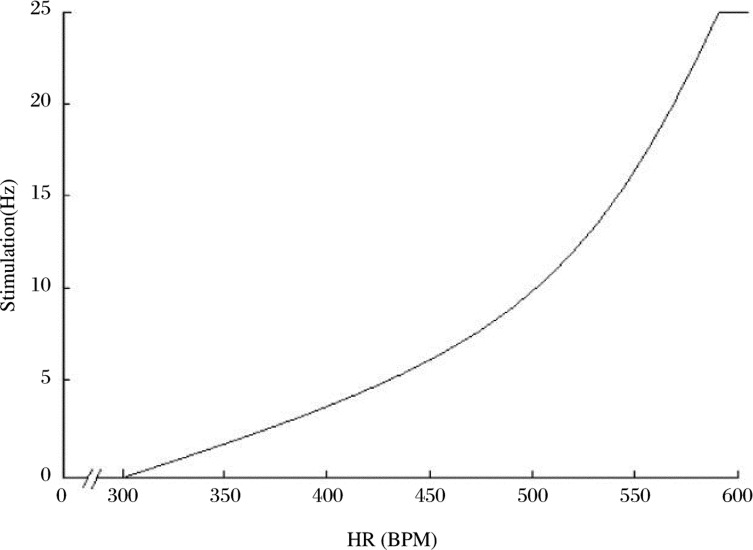
Relationship between HR and the frequency of stimulation in the chip systems.

The basic regulation principle of the chip system is simply expressed as the relationship between HR and frequency of stimulus. When the HR was between 300 and 580 bpm, the stimulus frequency increased following the elevation of the HR. The pattern of stimulus changing is represented in ***Fig. 4***. The increased stimulation of the vagus nerve consequently caused a decrease in the HR. When the HR exceeded 580 bpm, the frequency of stimulus reached its maximal value of 25 Hz. However when the HR was less than 300 bpm, the stimulation stops automatically (***Fig. 5***). In addition, the upper limit and lower limit of the relationship between stimulation and HR could be set by the master machine through the wireless communication.

#### The Master Machine

The master machine served as a data bridge between the slave machine and PC. It received data from the slave machine and then sent them to the PC user software. At the same time the user's command would be sent back to the slave machine. The user command included:

① Frequency of stimulation control;

② Stimulation starting point control (upper and lower limits of the HR stimulation relationship);

③ R wave recognition threshold control;

④ PWM stimulation duty cycle control;

⑤ Temporary data sending (the temporary data stored in the flash memory of the slave machine);

⑥ Temporary data clearance.

The RF link data were collected via a Nordic nRF24L01 transceiver on the master side. The transceiver was operated by a MSP430F169 microcontroller and the communication between the master machine and PC achieved by a USB bridge IC (FTDI FT245BM).

#### Wireless Communication

The Nordic nRF24L01 was chosen to deal with the wireless communication. It was a single chip 2.4 GHz transceiver with an embedded baseband protocol engine (Enhanced ShockBurst^TM^), designed for ultra low power wireless applications.

The main features of Enhanced ShockBurst^TM^ are as follow:

① 1 to 32 bytes dynamic payload length;

② Automatic packet handling;

③ Auto packet transaction handling;

④ Auto acknowledgement;

⑤ Auto retransmit;

⑥ 6 data pipe MultiCeiver^TM^ for 1:6 star networks

Importantly, the dynamic payload mode feature could achieve the half duplex communication between the slave and the master side. Therefore, without changing the mode from sending to receiving, the power consumption was ultra low during two-way communication. In addition, with the auto packet transaction handling feature the communication was quite safe and robust. One master machine could control 6 slave machines, thus the data from 6 different test animals could be collected simultaneously [17].

#### User Software

The whole system was configured with the software MD2000WL running on Windows-based PC's. The software of the MD2000WL data acquisition system (Nanjing Medical University) was developed by MFC, including data plotting, data filtering and data analysis. MD2000WL was based on the ordinary MD2000U system with the addition of the slave machine setting and control interface. The control function included stimulation starting point control (upper and lower limits of the heart rate stimulation relationship), flash data control, R wave recognition threshold control, and stimulation duty cycle control.

### Animals and Surgical procedure

Six male SD rats with normal HR weighing 400-500 g were used to test the system. All animals were obtained from Laboratory Animal Centers of Jiangsu Province and were handled according to the guidelines approved by the Experimental Animal Care and Use Committee of the Nanjing Medical University. Each rat was anesthetized with an intraperitoneal injection of chloral hydrate (0.4 g/kg). The right cervical vagus nerve was isolated and a pair of silver electrodes was placed around the nerve in preparation for the stimulations. Two silver detection electrodes were placed under the skin of the chest to acquire ECG signals with one at the cardiac apex and the other at the cardiac base. The main part of the chip was subcutaneously implanted into the abdomen with wires tunneled under the skin to connect with the stimulation electrodes and the detection electrodes. The rats were caged in a controlled temperature and humidity environment with a 12 h light/dark cycle. Standard laboratory chow and drinking water were available ad libitum.

### Protocols

1-3 h after the surgical process, rats recovered from anesthesia. We spent an additional 2 h allowing the animals to resume normal behavior, after which each rat was subjected to a 30-min regulation and 30-min recovery period. ECG monitoring lasted for 24 h after the surgery in order to test if the chip system and its wireless communication were dependable.

### Statistical Analysis

Differences among the three observations in the same animal were assessed by Student's paired *t* test using SPSS 16.0. The criterion for statistical significance was set at *P* < 0.05. All data are presented as mean ± SD.

## RESULTS

**Fig. 6:**
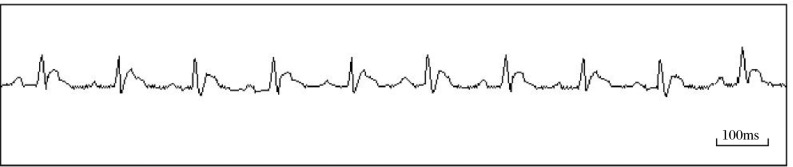
Representative ECG recording in conscious rats after the surgery

ECG signals were successfully collected during the experiment. R-R interval recognition accuracy was more than 95%. The restlessness of the rats gave rise to most errors in R-R interval recognition. An example of the ECG signals acquired is shown in ***Fig. 6***.

**Fig. 7:**
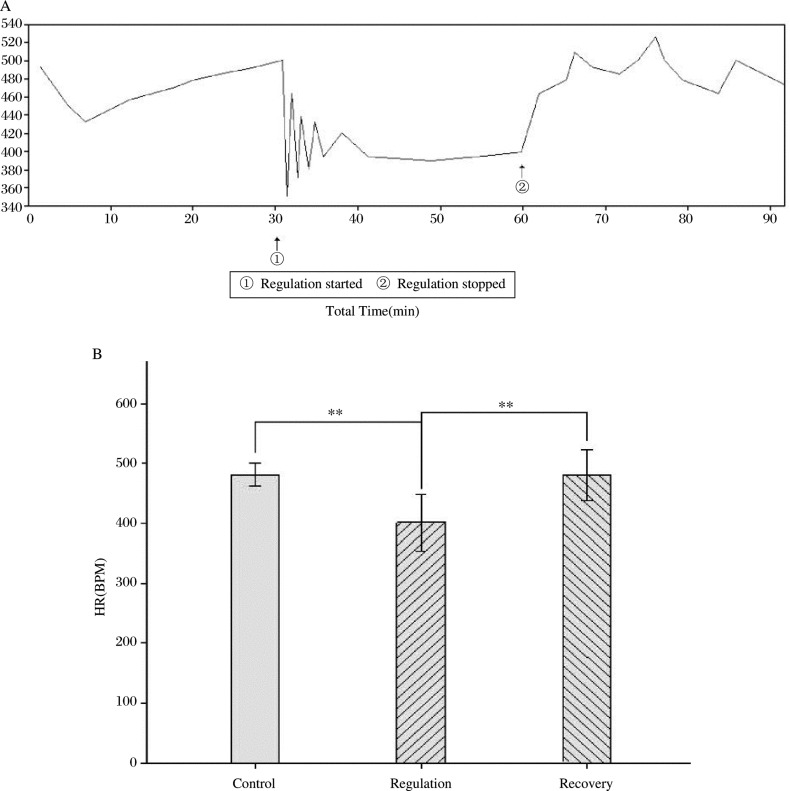
Representative changes (A) and statistical analysis (B) of HR recording during the periods of regulation and the recovery. A:representative changs of HR; B: statistical analysis of HR, **P < 0.01.

The HR was controlled successfully by the chip system. The change in HR during regulation and recovery is shown in ***Fig. 7***. During the regulation period, the HR was stably controlled at a lower level after an initial stage of fluctuation. After the regulation the stimulation was stopped and the HR returned to baseline in 1 min.

### HR Changes

The HR decreased significantly during the period of regulation compared with the control period (-79.3±34.5, *P* < 0.01, *n* = 6) and with the recovery period (-79. 0±28.8, *P* < 0.01, *n* = 6). There was no significant difference in HR between the control and the recovery periods (***Fig. 7B***).

## DISCUSSION

The primary finding of the present study was that a closed-loop implanted chip system used to control HR was successfully established in conscious animals. There is ample evidence that direct electrical stimulation of the vagus nerve can change HR in both animals and humans [10]–[11]. However, the use of an implanted chip to regulate HR at predetermined levels has not yet been reported. This study presents an example of using a closed-loop implanted chip system to artificially regulate the physiological function of an animal. We have successfully produced a Remote-controlled animal model for the control of visceral functions. It is more important to control HR in conscious rats than in anesthetized animals.

The chip system was based on the theory of establishing an artificial set point to take the place of the animal's own set point. ECG signals were recorded and converted to HR data through R-R interval recognition, and then the signals were sent back to the animals by means of stimulating the cervical vagus nerve. Whenever HR exceeded the set point, the stimulation to the vagus nerve would start. The higher the HR went, the stronger the stimulation generated. In this way, the closed-loop regulation maintained HR in a relatively narrow range, which was slightly higher than the artificial set point. It was found that there was a damped oscillation of HR after the regulation started, which might indicate that the chip regulation acted as a moderately damped negative feedback loop. When HR accelerated, the stimulation would become stronger as programmed, thereby reducing the HR. Until the regulation system of the body adapted to the chip system, HR changing became stable. Because of the complexity of the HR regulating mechanism, it is still not fully understood whether slowing of HR is mediated by activation of efferent vagus fibers or by sympathetic reflex inhibition through afferent fibers [10],[18]. The detailed physiological mechanism of such a damped oscillation is not well known.

The goal of closed-loop control of HR has been achieved and the system worked steadily without any unexpected problems. Nevertheless, improvement is required. Firstly, in contrast to anaesthetized animals, after such a surgery the conscious animals were agitated because of the pain and fear and their restlessness sometimes influenced our data collection. Although the R-R interval recognition was basically accurate because of the algorithm used, the electrodes fixation method and the ECG collection pattern still need to be improved. Secondly the size of the slave machine should be reduced in the future with a specific integrated circuit design so as to not overburden the test animals.

In summary, the chip system successfully regulated HR to an artificial set point in conscious rats, and this set point could be changed remotely. The present study laid a foundation for using the chip system to regulate physiological function and potentially to treat disease.
